# The function of phosphoglycerate mutase 1 (PGAM1) and its role in diseases

**DOI:** 10.3389/fmolb.2026.1842545

**Published:** 2026-08-17

**Authors:** Aihong Peng, Junqin Li, Huifang Liang, Yuanjun Yao, Kaiming Zhang

**Affiliations:** Shanxi Key Laboratory of Stem Cells for Immunological Dermatosis, State Key Breeding Laboratory of Stem Cells for Immunological Dermatosis, Institute of Dermatology, Taiyuan Central Hospital, Taiyuan, China

**Keywords:** diseases, inhibitors, metabolic, non-metabolic, PGAM1

## Abstract

Phosphoglycerate mutase 1 (PGAM1) is a pivotal metabolic enzyme during the process of glycolysis, catalyzing the interconversion between 3-phosphoglycerate and 2-phosphoglycerate. PGAM1 is mainly involved in cellular energy metabolism and biosynthesis, it is generally upregulated in numerous human cancers and promotes cell proliferation, invasion, and drug resistance. It serves as a significant biomarker for tumor diagnosis and a potential therapeutic target. In this paper, the basic structure and functions of PGAM1 and its family members, metabolic, non-metabolic activity and the regulatory mechanisms of PGAM1 and its correlation with diseases, as well as the latest progress of PGAM1 inhibitors in tumor diagnosis and treatment are introduced, aiming to provide new ideas for the targeted therapy of related diseases.

## 1 Introduction

Phosphoglycerate mutase (PGAM) was first discovered in yeast, and later it was found to be expressed in a variety of organisms such as rats, mice and humans through crystal structure determination and amino acid sequence analysis (Sharif et al., 2019; Wu et al., 2021). Studies have found that PGAM has five isoforms (PGAM1-5). Sequence alignment and crystal structure analysis show that PGAMs are highly conserved from bacteria to humans, and their functions are mainly characterized by glycolytic and non-glycolytic activities (Li et al., 2020a). PGAM1 involved in other metabolic processes through the transformation balance between 3-PG and 2-PG (Hitosugi et al., 2012). PGAM2 and PGAM1 have about 80% homology, and plays the same role in the glycolytic pathway as PGAM1 (Nukui et al., 2007). PGAM3 and PGAM4 are considered pseudogenes (Graa et al., 1992; Jin et al., 2013), and some studies have found that the essential amino acid sequence of PGAM3 active center is identical to that of PGAM1, and then it is speculated that PGAM3 may have similar enzymatic activity to PGAM1, but its function is still uncertain (Betran et al., 2002). Unlike other proteins in the family, PGAM5, as a mitochondrial protein phosphatase, plays an important role in the regulation of mitochondrial homeostasis (Takeda et al., 2009; Panda et al., 2016). When mitochondria were mildly damaged, PGAM5 activates mitochondrial biogenesis, thereby promoting the compensatory response of cells. However, when mitochondria were severely damaged, PGAM5 induces excessive mitochondrial division, disrupts mitochondrial movement, transmits apoptosis, necrosis or mitophagy death signals, and ultimately leads to the occurrence of cell death (Cheng et al., 2021).

**FIGURE 1:**
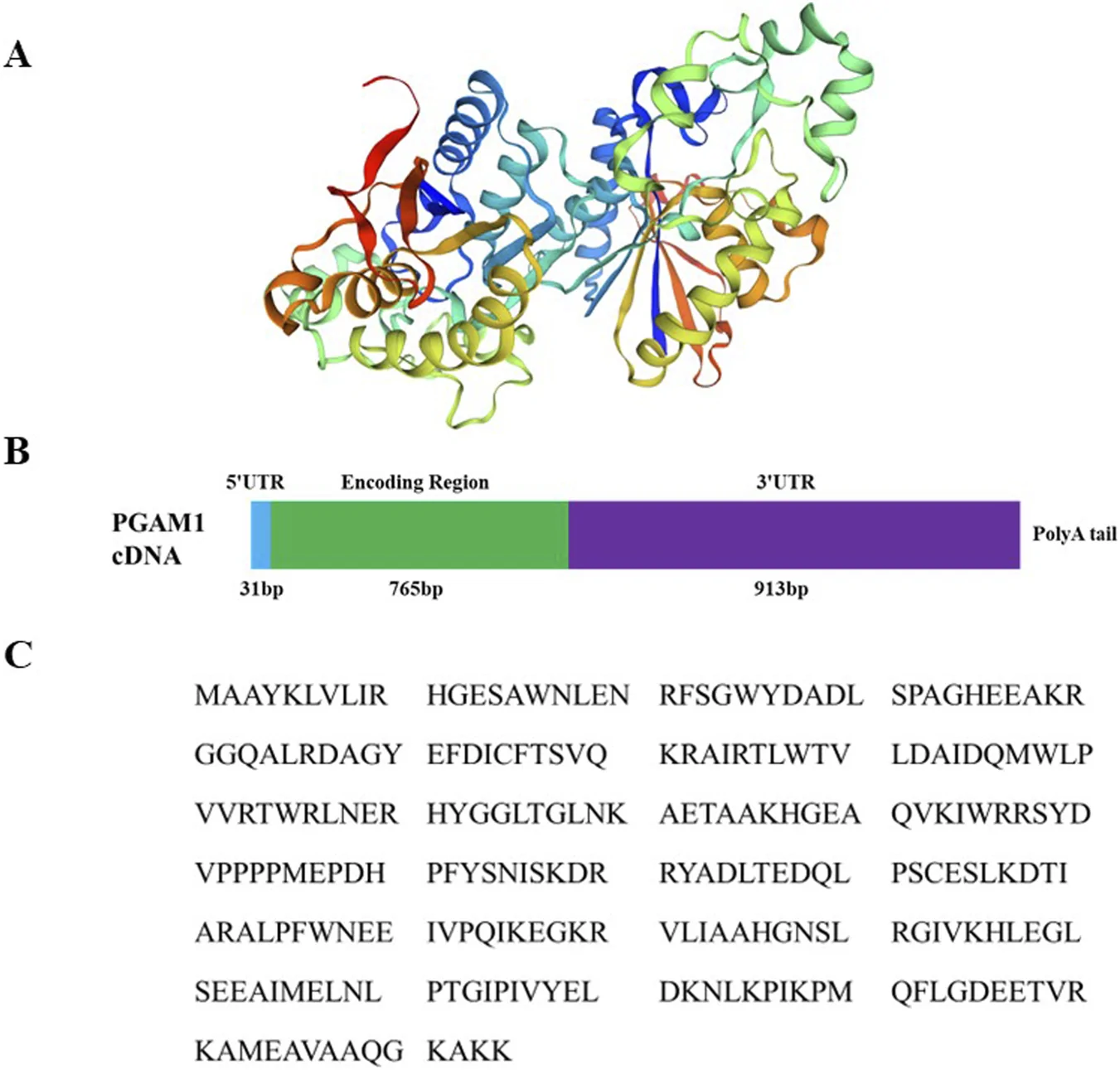
The crystal structure, the cDNA encoding and amino acid sequence of PGAM1. **(A)** The crystal structure of PGAM1. **(B)** The full-length of PGAM1 encoding cDNA. **(C)** Amino acid sequence of *Homo sapiens*’s PGAM1.

PGAM1 belongs to the phosphoglycerate mutase family, which can be divided into monophosphoglycerate mutase (mPGAM) and bisphosphoglycerate mutase (BPGAM). mPGAM mainly catalyzes the interconversion of 3-PG and 2-PG, whereas the conversion of 1, 3-bisphosphoglycerate (1, 3-BPG) to 2, 3-bisphosphoglycerate (2, 3-BPG) in the presence of 3-PG is catalyzed by BPGAM (Locasale et al., 2011; Fothergill-Gilmore and Watson, 1989). mPGAM also has a phosphatase activity of 2, 3-BPG, but this activity is much less than the normal mutase activity (Rodwell et al., 1957). In addition, mPGAM can be further divided into two categories: the ones dependent on 2, 3-BPG as a cofactor (dPGAM) and the ones independent of 2, 3-BPG (iPGAM) (Hitosugi et al., 2012). Previous studies have shown that only dPGAM and BPGAM are present in mammals, have kinetic and structural similarities and are considered to be homologous structures (Rose and Dube, 1978; Grisolia and Cleland, 1968). For example, dPGAM is involved in three catalytic reactions: the reversible conversion of 3-PG to 2-PG (Sasaki et al., 1976; Rose and Dube, 1976), the phosphatase reaction in which 2, 3-BPG is converted to PG (Rose and Dube, 1978; Sasaki et al., 1971), and the synthase reaction in which 1, 3-BPG is converted to 2, 3-BPG, which is similar to BPGAM. Human PGAM is a dimeric protein with two distinct subunits, namely, brain PGAM1 (B-PGAM) and muscle PGAM2 (M-PGAM). In adult mammals, the two subunits can combine to form three isozymes: two homodimers (B-PGAM/B-PGAM, M-PGAM/M-PGAM) and one heterodimer (B-PGAM/M-PGAM) (Fothergill-Gilmore and Watson, 1989). Among them, the homodimeric BB-PGAM (Figure 1A) was originally isolated from the brain (called PGAM1 in humans) and is expressed in breast, kidney, liver, and other tissues (Ren et al., 2010; Evans et al., 2007). Heterodimeric BM-PGAM is mainly expressed in the heart (Durany and Carreras, 1996). However, the homodimer MM-PGAM2 (human PGAM2) is a type that is mainly present in mature muscle skeletal and cardiac cells (Omenn and Cheung, 1974).

## 2 Basic structure of PGAM1

The human PGAM1 gene is located at chromosome 10q25.3 and encodes a 1709 bp cDNA, which encodes a 254 amino acid long chain protein with a molecular weight of 28,784 Da, including a 31 bp 5′ untranslated region (5′UTR), a 765 bp coding region and a 913 bp 3′ untranslated region (3′UTR) extending to the tail of polyA (Blouquit et al., 1988) (Figure 1B). The cDNA of PGAM2 encodes a 253 amino acid and contains a short 3′ UTR (Shanske et al., 1987) (Figure 1C). In addition to the similarity of amino acid residue number and molecular weight, the content of acidic and basic amino acid residues of PGAM1 and PGAM2 is quite different, and the isoelectric point values are different, which are 7.0 and 10.0, respectively (Sakoda et al., 1988). The PGAM2 gene has only one copy in the human genome, whereas the PGAM1 gene has multiple copies. Therefore, PGAM1 is a precursor of PGAM2 (Nir et al., 1977; Betran et al., 2002; Hong and Lee, 2018) (Table 1).

**TABLE 1:** Differences between five isoforms of PGAM enzymes.

No.	Enzyme	Chromosome	Amino acids	Subcellular localization	References
1	PGAM1	10	254	Nucleoplasm	Sakoda et al. (1988)
2	PGAM2	7	253	Cytosol	Nir et al. (1977)
3	PGAM3	Pseudo gene			Hong and Lee (2018)
4	PGAM4	X	255	Nucleoplasm	Jin et al. (2013)
5	PGAM5	12	290	Mitochondria	Vander et al. (2009)

## 3 The metabolic activity of PGAM1

Glycolysis is the core pathway of glucose metabolism ubiquitous, which is the first step of glucose metabolism in all living cells. It refers to the process in which glucose is gradually decomposed into pyruvate in the cytoplasm by a series of enzymes without the participation of oxygen, and a small amount of adenosine triphosphate (ATP) and nicotinamide adenine dinucleotide (NADH) are produced (Vander et al., 2009). It is not only the common initial step of aerobic and anaerobic respiration, but also can quickly supply energy to cells, provide key intermediates for subsequent metabolic pathways, and provide precursors for the synthesis of biomacromolecules. It is an important link to maintain the basic energy demand of cells and substance metabolism homeostasis (Xu et al., 2017).

**FIGURE 2:**
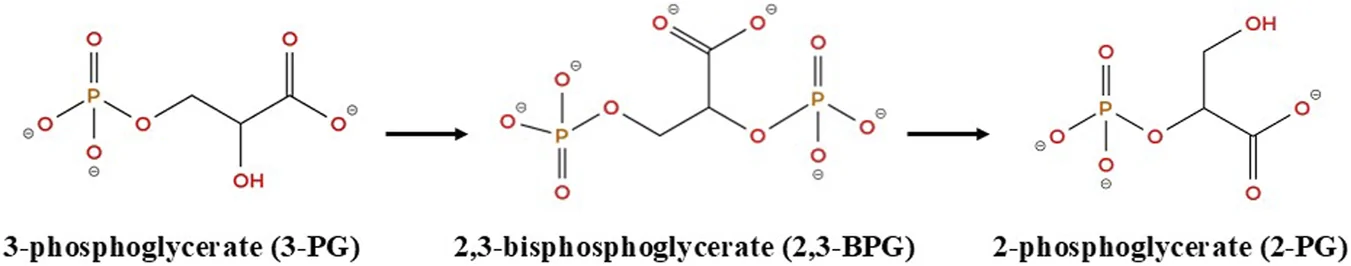
PGAM 1 reaction and structural representation of 3-PG, 2,3-BPG, and 2-PG.

PGAM1 is catalyzes the reversible conversion of 3-phosphoglycerate (3-PG) to 2-phosphoglycerate (2-PG), forming 2, 3-diphosphoglycerate (2, 3-BPG) as an intermediate (Buhrens et al., 2009) (Figure 2). The activity of PGAM1 is dependent on the phosphorylation of the active site histidine (H11). Firstly, H11 of PGAM1 is phosphorylated by a phosphate group. Then, 3-PG is immersed in the catalytic pocket of the enzyme, and the phosphate group in H11 is transferred to the C-2 of 3-phosphoglycerate, promoting the formation of 2, 3-BPG in the catalytic pocket, and the product 2, 3-BPG reneweds the phosphorylation of H11 by trapping the C-3 phosphate group on 2, 3-BPG. Under the certain conditions, fructose-1, 6-diphosphate (FBP) or 1, 3-diphosphoglycerate (1, 3-diphosphoglycerate) can also act as phosphate donors (Hitosugi et al., 2013; Liu et al., 2017; Oslund et al., 2017). It is well known that glycolysis provides intermediates for biosynthesis. PGAM1 participates in these processes through its mutant enzymatic activity (Zhang et al., 2017a). It was shown that PGAM1 synchronizes glycolysis and anabolic synthesis because the intracellular substrate 3-phosphoglycerate is known to act as an inhibitor of 6-phosphoglycerate dehydrogenase and reduces the nucleotide synthesis reaction in the oxidative pentose phosphate flux. 1, 3-diphosphoglycerate is a highly active glycolytic intermediate involved in ATP production through direct phosphorylation of PGAM1 by phosphoglycerate kinase. After PGAM1 is phosphorylated by 1, 3-BPG, the production of 2, 3-BPG is promoted by transferring its phosphate group to glyceric acid monophosphate (Hitosugi et al., 2013). 1, 3-BPG phosphorylates the PGAM1, but when BPGAM is deleted, PGAM1 is in a hypophosphorylated state, and intracellular level of 3-PG is increased, but the accumulation of 3-PG is not sufficient to block the oxidative pentose phosphate pathway (PPP) (Liu et al., 2017). Since 2-PG activates phosphoglycerate dehydrogenase can affect the serine pathway, the levels of phosphoserine and serine were greatly increased. All these metabolic alterations occurred in the absence of BPGAM. The increased expression of active BPGM completely liberated the metabolic phenotype. In contrast, PGAM1 could not fully compensate for the effects caused by BPGAM deletion, because the elevated activity of PGAM1 may not be capable to reduce the levels of monophosphoglycerate and phosphoserine (Oslund et al., 2017).

## 4 The non-metabolic activity of PGAM1

In addition to metabolic activity, PGAM1 mediates several non-metabolic activities. Zhang et al. demonstrated that PGAM1 directly interacts with α-smooth muscle actin (ACTA2) in a non-metabolically dependent manner, without being dependent on its metabolic activity. ACTA2 gene encoded a globular actin, a component of filamentous actin (F-actin), which is involved in cytoskeleton remodeling and regulates cell movement. Its dynamic assembly and dissociation mediate the assembly of actin filaments, cell movement, and the migration of cancer cells, and this interaction is not related to its metabolic activity (Olson and Sahai, 2009). The C-terminal amino acids 201-210 are essential for the binding of PGAM1 to the cytoskeletal protein ACTA2. This interaction does not require the PGAM activity. To exclude the influence of glycolytic enzymes, such as hexokinase 2 (HK2), pyruvate kinase M2 (PKM2), lactate dehydrogenase A (LDHA), and pyruvate dehydrogenase kinase 1 (PDK1), were individually depleted in MDA-MB-231 cells (Zhang et al., 2017a). Deletion or knockout of PGAM1 alters the ratio of G-actin to F-actin, leading to cytoskeletal remodeling in breast cancer cells, and thereby reducing its metastatic ability. Combined survival analysis showed that breast cancer patients with high expression of PGAM1-ACTA2 had a poorer prognosis, indicating that PGAM1 mediates tumorigenesis and metastasis in a non-metabolically dependent manner. It is an important molecular target related to tumor metastasis (Zhang et al., 2017a). Zhang et al. (2017b) found that after knocking down PGAM1 in Cal27 and HN12 cells, the cell migration ability was significantly reduced, and at the same time, the signaling pathway molecules such as proto-oncogene c-SRC (SRC), focal adhesion kinase (FAK), and Paxillin were also inhibited. Liu X. L. et al. (2018) found that PGAM1 promotes epithelial-mesenchymal transition (EMT) in pancreatic ductal adenocarcinoma (PDAC) cell lines by regulating the Wnt/β-catenin pathway. Silencing PGAM1 reduced the proliferation of Aspc-1 and Panc-1 cells, and led to S-phase arrest, but did not affect cell apoptosis. The cell migration and invasion abilities were also significantly reduced, and were not related to proliferation. PGAM1, as a downstream target of the phosphatidylinositol-4, 5-diphospho-3-kinase/protein kinase-B/mammalian target of rapamycin (PI3K/AKT/mTOR) pathway, is regulated by this pathway and has a positive interaction relationship with HIF-1α. In addition, PGAM1 can assemble phosphoglycerate kinase 1/PGAM1/α-enolase/ nuclear paraspeckle assembly transcript 1 (PGK1/PGAM1/ENO1/NEAT1) complex, and improving glycolysis efficiency (Park et al., 2021). Therefore, PGAM1 has potential transfer ability, and its transfer pathway is not related to metabolism (Zhang et al., 2017a).

PGAM1 exerts its biological functions through two distinct mechanisms-metabolic activity and non-metabolic activity-collectively determined by the protein’s unique three-dimensional structure, subcellular localization, post-translational modifications, and dynamic regulation of intracellular metabolic microenvironments (Fushinobu et al., 2011; Wiśniewski et al., 2022). PGAM1 possesses spatially separated catalytic active sites and protein interaction interfaces, which serve as the structural basis for its dual functions (Wiśniewski et al., 2022). In the cytoplasm, abundant glycolytic substrates stabilize PGAM1 homodimers, and the catalytic pocket enables PGAM1 to exert its enzymatic activity to sustain basal cellular glycolysis. Under the conditions of DNA damage, hypoxia or tumor stress, PGAM1 undergoes ubiquitination modification and translocates to the nucleus. The low-glucose microenvironment drives the dissociation of PGAM1 homodimers into monomers in the nucleus, resulting in the collapse of the catalytic site conformation and the loss of metabolic activity. The independent protein interaction region is fully exposed, mediating chromatin binding, DNA repair, and transcriptional regulation of target genes, which are gene regulatory processes that do not rely on enzymatic activity. The acetylation at the lysine K100 site is the core molecular switch that regulates this functional transition (Zhang et al., 2020). The modification is enriched at the dimer binding interface, breaking the intermolecular hydrophobic forces, promoting homodimer dissociation into monomers, directly causes the loss of enzyme activity, activates non-enzymatic functions, and enhances protein-binding capacity. In contrast, SIRT1-mediated deacetylation modification stabilizes the dimeric conformation and maintain its metabolic enzyme activity (Hallows et al., 2012). Meanwhile, the intermediate products of glycolysis combine with catalytic pockets to stabilize the dimeric structure, maintain enzymatic activity and inhibit nuclear translocation. When hungry, the glucose metabolic substrate is exhausted and cannot support dimer assembly. PGAM1 undergoes monomerization and translocates into the nucleus, and non-enzymatic functions become dominant. The cellular metabolic state directly feedback regulates PGAM1, forming a metabolism-signal closed loop (Hitosugi et al., 2012). From an evolutionary perspective, PGAM1’s dual-function design allows it to link energy metabolism with gene homeostasis regulation, reduces genomic coding burden. In malignant tumors, the coordinated activation of both enzymatic metabolic function and nuclear non-enzymatic function drives tumor proliferation and invasion, which also explains the limited efficacy of current inhibitors targeting only the catalytic site (Hitosugi et al., 2012).

## 5 Mechanisms of multilevel regulation of PGAM1

The regulatory mechanisms of PGAM1 mainly include post-transcriptional regulation, post-translational modification regulation and signaling pathway regulation. In mammals, the cofactor 2, 3-BPG transfers the phosphate group at the C3 position to the H11 site of PGAM1, and phosphorylates it to upregulate the enzymatic activity of PGAM1 (Grisolia and Cleland, 1968). The Nm23-H1/ glyceraldehyde 3-phosphate dehydrogenase (GAPDH) complex phosphorylates and activates PGAM1 at the H10. The substrate of non-metastatic protein 23 homolog 1 (Nm23-H1), deoxycytidine triphosphate (dCTP), is also found to be a PGAM1 activator *in vitro* (Engel et al., 1998; Engel et al., 2004). Its activity was upregulated by the receptor tyrosine kinase FGFR1-dependent tyrosine phosphorylation and Y26 phosphorylation of PGAM1 (which changes the conformation of PGAM1 by releasing a negatively charged E19 and promote 2,3-BPG binding). Moreover, Y26 phosphorylation is an important activation mechanism of PGAM1 in leukemia and other solid tumors (Hitosugi et al., 2013).

The research showed that PGAM1 is positively regulated by the PI3K/AKT/mTOR pathway, and is mutually positively regulated with hypoxia inducible factor-1α (HIF-1α) (Liu X. L. et al., 2018). The PI3K/AKT/mTOR pathway is a key regulator of aerobic glycolysis, and abnormal activation of this pathway increases the translation and transcription of HIF-1α. HIF-1α, as a transcriptional activator of PGAM1, plays a key role in mTOR-mediated PGAM1 expression. Meanwhile, the AKT signaling cascade was independent of oxygen levels and further enhances its regulatory effect on PGAM1 by stabilizing and transactivating HIF-1α, and there were mutual positive regulation between PGAM1 and HIF-1α (Sun et al., 2018; Singh et al., 2017). In addition, HIF-1α promotes tumor glycolysis by interacting with PKM2. PGAM1, as an important enzyme of the Warburg effect (Semenza, 2007; Luo and Semenza, 2011; Yang G. J. et al., 2021), is regulated by HIF-1 (Jiang et al., 2014), and it acts as a downstream effect protein of mTOR signaling. The mTOR-PGAM1 signaling cascade may be involved in the tumor Warburg effect. In non-small cell lung cancer (NSCLC), PGAM1 inhibition reduced mTOR-dependent glycolysis and tumorigenesis. The level of PGAM1 was positively correlated with mTOR activity, and was a positive biomarker for poor prognosis. Meanwhile, PGAM1 plays an important role in mTOR-mediated tumor growth (Sun et al., 2018). Zhang et al. discovered that targeting PGAM1 not only inhibits the growth of hepatocellular carcinoma (HCC) cells by promoting ferroptosis through energy stress/ROS-dependent AKT/ lipocalin (LCN2) axis degradation, but also downregulates programmed death receptor 1 ligand 1 (PD-L1), thereby enhancing CD8+T cell-mediated anti-tumor immunity. Moreover, it acts synergistically with anti-PD-1 immunotherapy to optimize the immune microenvironment (Zheng et al., 2023). Several studies have shown that PGAM1 is overexpressed, negatively correlated with p53 activity in cancer cells, and negatively regulated cellular glycolysis (Kondoh et al., 2005; Bensaad et al., 2006; Madan et al., 2011), suggesting that loss of tumor suppressor gene tumor protein p53 (TP53) promotes PGAM1 expression in cancer cells (Kondoh et al., 2005). Wild-type p53 reduces the expression level of PGAM1, and p53 mutation promotes the immortalization of mouse embryonic fibroblasts by affecting PGAM level and glycolysis (Bensaad et al., 2006; Madan et al., 2011; Mau´es et al., 2021).

In addition to being regulated at the transcriptional and translational levels, PGAM1 is also regulated by epigenetic modifications such as ubiquitination, acetylation, and lysine succinylation. In human colorectal cancer and colon cancer, the level of PGAM1 monubiquitination is significantly increased, and the non-covalent complexes containing ubiquitin and PGAM1 also show a similar trend. The abundance of PGAM1 ubiquitination gradually increases during the staging and progression of colon cancer, suggesting that it may be related to tumor progression. However, the research on the ubiquitination modification sites and specific mechanisms of PGAM1 is still not in-depth (Usuba et al., 2001). Acetylation/deacetylation is a common modification of most enzymes in glycolysis and is associated with aging and age-related diseases (Zhao et al., 2010). PGAM1 is also regulated in this way, and the nicotinamide adenine dinucleotide (NAD+) -dependent protein deacetylase Sirt1 affects metabolic pathways at the transcriptional level, regulates gluconeogenesis, promotes β-oxidation and inhibits adipogenesis (Finkel et al., 2009; Haigis and Sinclair, 2010). Further research found that the acetylation level and activity of PGAM1 were negatively correlated with the activity of Sirt1 (Hallows et al., 2012). Sirt1-mediated deacetylation of PGAM1 upregulates the level of 3-PG intermediates, reduces the level of 2-PG, and ultimately blocks the anabolic reactions. It was found that the activity of Sirt1 was enhanced and the acetylation level and activity of PGAM1 are decreased under glucose restriction. However, it remains to be further confirmed whether Sirt1 dysfunction is directly related to tumor glycolysis or promotes the Warburg effect. In addition, HAT1, a histone acetyltransferase, can act as a succinyltransferase in HepG2 cells, enhancing its enzymatic activity and stimulating glycolytic flux by catalyzing lysine succinylation at K99 of PGAM1 (Mu et al., 2021; Yang G. et al., 2021).

## 6 The role of PGAM1 in cancer

We systematically categorized all diseases covered in existing PGAM1 research and clearly pointed out that cancers (including breast cancer, hepatocellular carcinoma, lung cancer, glioblastoma, etc.) are the most extensively investigated disease for PGAM1, PGAM1 drives activation of the oxidative pentose phosphate pathway (PPP) and initiates the serine biosynthesis axis. Metabolic programs are indispensable for sustaining cancer in cell proliferation and tumorigenesis. Accordingly, abundant research has sought to exploring the associations between PGAM1 and the clinical characteristics in multiple human cancers.

### 6.1 PGAM1 in lung cancer

Lung cancer is one of the leading causes of cancer-related deaths worldwide, with non-small cell lung cancer (NSCLC) accounting for approximately 85% of all lung cancer cases (Zheng et al., 2022). PGAM1 has been the most extensively studied in NSCLC (Zhou et al., 2025; Liang et al., 2021; Li et al., 2020b). Emerging evidence indicates that glycolysis-derived lactate can mediate protein lactylation of the m^6^A methyltransferase METTL14. METTL14 catalyzes m^6^A methylation modification on PGAM1 mRNA, prolonging its half-life and upregulating PGAM1 protein expression, which in turn continuously enhances the aerobic glycolysis of tumor cells and ultimately facilitates the proliferation of non-small cell lung cancer cells. Collectively, these findings reveal a novel molecular mechanism by which lactylation, RNA m^6^A epigenetic modifications, and tumor metabolic reprogramming synergistically regulate the malignant progression of lung cancer, providing new targets for prognostic biomarkers and metabolism-targeted therapy of lung cancer (Wang and Liu, 2026). Huang et al. demonstrated that PGAM1 is highly expressed in NSCLC. Inhibiting PGAM1 exerts a dual anti-tumor effect: on one hand, it inhibits the conversion of 3-PG to 2-PG, disrupts redox homeostasis, activates the JNK/c-Jun pro-apoptotic pathway, and inhibits the AKT/ERK pro-proliferative pathway. This effectively arrests NSCLC cell proliferation and overcomes erlotinib resistance. On the other hand, it disrupts the protein binding of PGAM1-ACTA2 (Huang et al., 2019c), thereby weakening tumor invasion and metastasis. It is clear that PGAM1 is a highly promising therapeutic target for NSCLC, providing novel strategies and lead compounds for the development of metabolism-targeted anti-lung cancer drugs. Emerging evidence has demonstrated that the allosteric inhibitor HKB99 of PGAM1 can effectively reverse EGFR-targeted resistance. Mechanistically, HKB99 suppresses PGAM1 activity and downregulates IL-6 secretion, thereby blocking the activation of the downstream JAK2/STAT3 signaling pathway, inhibiting the proliferation, invasion and glycolytic phenotype of resistant cells. *In vivo* animal experiments have verified that this inhibitor restores the sensitivity of resistant tumors to EGFR-targeted drugs, revealing the PGAM1-IL-6/JAK2/STAT3 axis is the key pathway for EGFR-TKI resistance (Liang et al., 2023). This provides a theoretical rationale and novel therapeutic strategies for overcoming drug resistance by combining PGAM1 inhibitors with EGFR-targeted drugs in clinical.

### 6.2 PGAM1 in breast cancer

Breast cancer (BC) is one of the most common causes of cancer-related deaths among women. There were approximately 2.3 million breast cancer worldwide in 2020, accounting for 11.7% of all cancer cases (Sung et al., 2021). The mRNA and protein expression levels of PGAM1 in breast cancer and triple-negative breast cancer tissues were significantly higher than those in normal tissues. PGAM1 promotes the progression of breast cancer through multiple signaling pathways and has become a crucial potential target for targeted therapy. One is that the expression of argininosuccinate synthase 1 (ASS1) is negatively regulated by the cyclic adenosine monophosphate/ adenosine 5′-monophosphate-activated protein kinase/ CCAAT/enhancer-binding protein beta (cAMP/AMPK/CEBPB) axis. Knockdown of PGAM1 increases the intracellular cAMP level, activates the AMPK pathway to suppress transcription factor CEBPB, and ultimately upregulates the tumor-suppressive molecule ASS1, thereby restrains malignant phenotypes of breast cancer cells including proliferation, invasion, and epithelial-mesenchymal transition (EMT). The PGAM1-ASS1 regulatory loop together with its upstream and downstream cAMP/AMPK/CEBPB cascade have clarified a novel oncogenic mechanism underlying metabolic reprogramming in breast cancer (Liu et al., 2022).

Second, PGAM1 activates the Wnt/β-catenin signaling pathway to promote the occurrence and development of breast cancer, promotes nuclear translocation of β-catenin and upregulates the expression of downstream oncogenic target genes, enhances the proliferation, invasion and migration capacities of breast cancer. Inhibition of PGAM1 blocks aberrantly activated Wnt/β-catenin signaling, reverses the malignant phenotypes of breast cancer, and simultaneously induces cell apoptosis and cell cycle arrest. These findings reveal that the PGAM1-Wnt/β-catenin regulatory axis is a key molecular mechanism driving the initiation and progression of breast cancer (Wang et al., 2025). Studies have shown that triple-negative breast cancer accounts for approximately 15% of all breast cancer cases. PGAM1 inhibition reshapes the tumor microenvironment of triple-negative breast cancer and in synergy with anti-PD-1 immunotherapy to enhance the anti-tumor effect, The combination significantly strengthens suppression of triple-negative breast cancer, offering a novel therapeutic strategy that integrates metabolic targeting with immune inhibitors (Zhang et al., 2024a). Zhang et al. have demonstrated that PGAM1 promotes the generation of immunosuppressive M2-type tumor-associated macrophages in the tumor microenvironment, reshaping the immunosuppressive microenvironment of breast cancer. This mechanism reveals a novel oncogenic role of PGAM1 in tumor immune regulation and provides a new therapeutic target for combined metabolic and immune targeting in breast cancer (Zhang et al., 2024b).

### 6.3 PGAM1 in hepatocellular carcinoma

Hepatocellular carcinoma (HCC) has a poor prognosis due to its high recurrence rates, late-stage diagnosis, and resistance to existing therapies (Sung et al., 2021; Luo et al., 2024). PGAM1 is a key enzyme in glycolysis and is upregulated in HCC and other cancers. Its overexpression is associated with poor prognosis and aggressive tumor behavior (Hitosugi et al., 2013; Zheng et al., 2023; Yang et al., 2022). The underlying mechanisms of combining immunotherapy with molecular targeted therapies in hepatocellular carcinoma (HCC) remain unclear. Through bioinformatics screening of multiple HCC datasets, discovered that PGAM1 is a novel immune-metabolic target. Mechanistically, inhibiting PGAM1 induces energy stress, inactivates the reactive oxygen species (ROS)-mediated AKT pathway, downregulates lipocalin (LCN2), thereby inducing triggers ferroptosis in HCC cells, and simultaneously reduces the expression of PD-L1 in tumor cells and promotes CD8^+^ T cells infiltration. Furthermore, the allosteric inhibitor KH3 exhibits synergistic efficacy with anti-PD-1 therapy, targeting PGAM1 represents a dual therapeutic strategy for HCC that combines tumor killing and immune improvement (Zheng et al., 2023). Zhu et al. (Zhu and Lu, 2024) found that astragaloside IV (AS-IV) could reduce the viability and glycolytic level of HCC cells and inhibit tumor growth in tumor-bearing mice by down-regulating the expression of KAT2A and inhibiting the succinylation of PGAM1 at the K161 site. This suggests that AS-IV may be a potential drug for the treatment of HCC. Research has found that the regulatory factor X6 (RFX6) is highly expressed in HCC tissues. RFX6 upregulates the expression of PGAM1, enhancing the aerobic glycolysis and promoting the proliferation and metastasis of HCC cells. Conversely, knocking down RFX6 downregulates the expression of PGAM1, inhibits glycolysis and blocks the malignant progression of HCC, confirming that the RFX6-PGAM1 axis is a critical pathway for regulating the metabolic reprogramming and driving tumor growth and metastasis of HCC (Qiu et al., 2023).

### 6.4 PGAM1 in pancreatic cancer

Pancreatic cancer is one of the most aggressive tumors, with a 5-year survival rate of approximately 8% (Atay, 2020). In pancreatic adenocarcinoma (PAAD), circRNA PGAM1 is abnormally overexpressed. It activates the AKT/mTOR signaling pathway. By continuously activating this oncogenic pathway, regulating the expression of downstream related effector molecules, enhancing the migration and invasion abilities of PAAD. Conversely, silencing circRNA PGAM1 inhibits the AKT/mTOR signaling pathway and reverses the malignant invasion and migration phenotype of tumor cells. It is clear that circRNA PGAM1 mediates the invasion and metastasis of pancreatic cancer by activating the AKT/mTOR pathway (Wang et al., 2024). In pancreatic ductal adenocarcinoma (PDAC), abnormal activation of the PI3K/Akt/mTOR pathway upregulates the expression of its downstream target PGAM1. The overexpression of PGAM1 reshapes glycolytic metabolism, induces epithelial-mesenchymal transition (EMT), alters the expression of EMT markers, including E-cadherin, Vimentin, and N-cadherin, weakens intercellular adhesion, thereby enhancing the migratory and invasive capabilities of tumor cells. Knocking down PGAM1 reverses the pro-metastatic effects triggered by the activation of this pathway. Collectively, these findings establish the PI3K/Akt/mTOR-PGAM1 regulatory axis, suggesting that PGAM1 serves as a potential therapeutic target for metastatic and treatment of PDAC (Liu X. L. et al., 2018).

### 6.5 PGAM1 in other cancers

Glioblastoma (GBM) is one of the most lethal brain tumors (Magne et al., 2021). Ubiquitin-conjugating enzyme E2S (UBE2S) is overexpressed in glioblastoma and inhibits the degradation of PGAM1 by interacting with Otubain-2, increases its protein level, promotes DNA repair and reduces cell apoptosis, and reducing the sensitivity of GBM cells to temozolomide (TMZ). Targeting UBE2S is expected to become a potential strategy for improving the chemotherapy effect of glioblastoma (Lin et al., 2025). Furthermore, PGAM1 is associated with the grading of glioblastoma, and promotes the migration, proliferation and invasion of glioblastoma (Xu et al., 2016; Liu Z. G. et al., 2018; Carvalho et al., 2020). Exosomal PGAM1 levels in the plasma of patients with metastatic prostate cancer (PCa) is significantly increased, promoting the invasion of PCa cells into pseudopodia formation, transporting human umbilical vein endothelial cells (HUVECs) to bind with γ-actin to promote podosome formation and neovascular sprouting in HUVECs, suggesting that exosomal PGAM1 promotes angiogenesis and serves as a liquid biopsy marker for PCa metastasis (Luo et al., 2023). Wen et al. conducted a comprehensive analysis of bioinformatics, animal experiments, and clinical specimen validation, and found that the expression of PGAM1 is closely related to the disease progression of renal clear cell carcinoma and the remodeling of the immune microenvironment (especially macrophage infiltration), and its inactivation enhanced the sensitivity of renal cancer cells to specific small molecule drugs (Wen et al., 2023).

**FIGURE 3:**
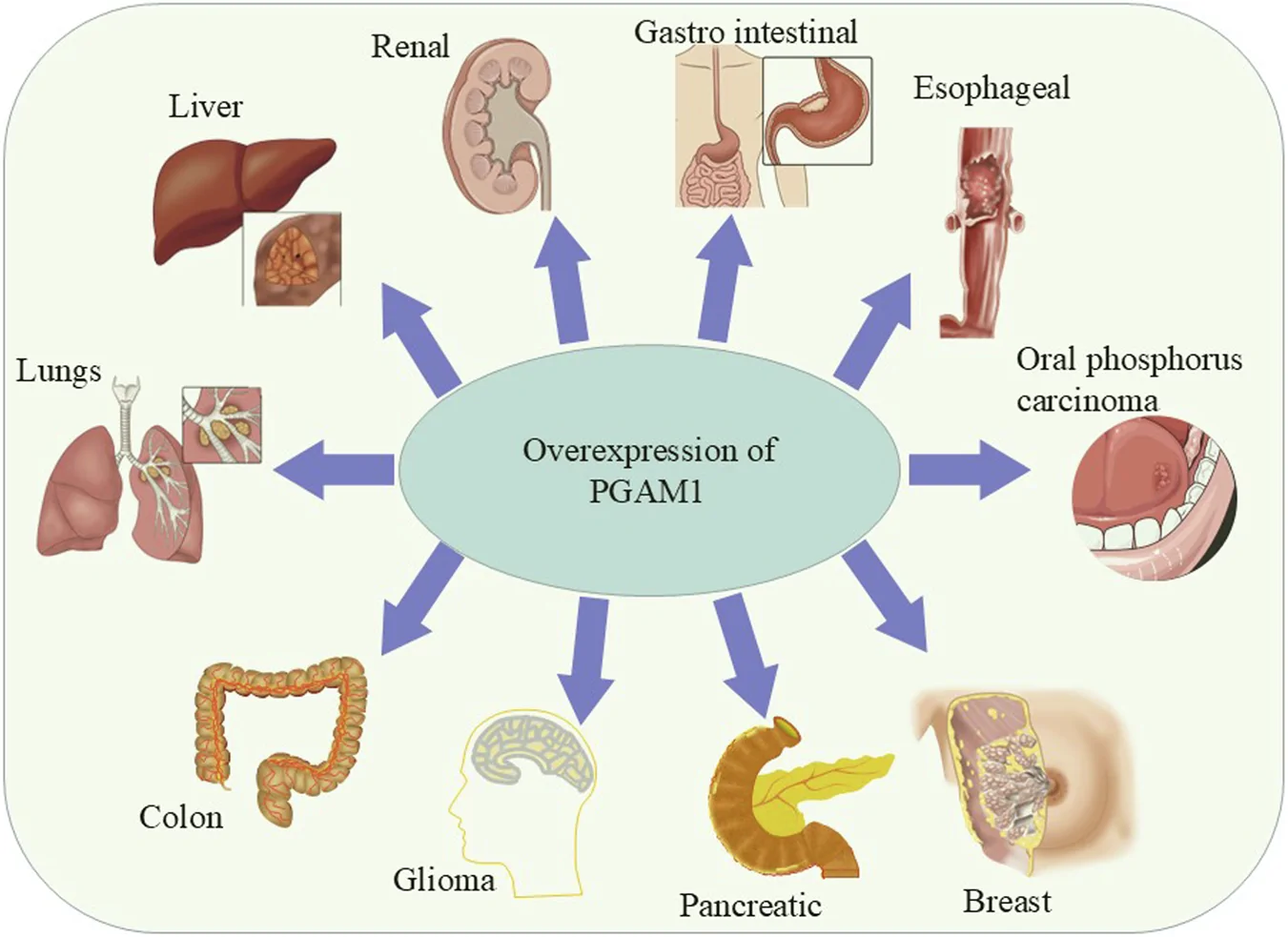
Overexpression of PGAM1 in different cancers.

Furthermore, studies have shown that PGAM1 is also overexpressed in oral squamous cell carcinoma (OSCC) (Zhang et al., 2017b), colorectal cancer (Liu et al., 2008) and urothelial bladder cancer (Table 2; Figure 3) (Peng et al., 2016).

**TABLE 2:** Targeting PGAM1 for cancer therapy: impact on tumor proliferation and metastasis.

Involved organ	PGAM1 inhibitor	Inhibit tumor proliferation	Inhibit tumor metastasis	Signal pathway	References
Breast cancer	MJE3	+	—	—	Evans et al. (2007), Evans et al. (2005)
	PGMI-004A	—	-	—	Hitosugi et al. (2012)
	siRNA	—	+	ACTA2	Hitosugi et al. (2012), Olson and Sahai (2009)
	shRNA	+	+	Wnt-β-catenin; cAMP-AMPK-CEBPB	Li et al. (2017), Wang et al. (2018)
	Xanthone derivatives	+	—	—	Wang et al. (2018)
HCC	shRNA	+	—	—	Ren et al. (2010)
	KH3	+	+	AKT-LCN2-ferroptosis; PD-L1	Luo and Semenza (2011), Huang et al. (2019b)
NSCLC	PGMI-004A	+	—	—	Hitosugi et al. (2012)
	HKB99	+	+	JNK-c-JUN; ACTA2; PAI-2	Liu et al. (2008)
	EGCG	+	—	—	Li et al. (2017)
	Xanthone derivatives	+	—	—	Wang et al. (2018)
	shRNA	+	—	PI3K-AKT-mTOR	Hallows et al. (2012)
Glioblastoma	siRNA	+	+	—	Xu et al. (2016)
PDAC	KH3	+	—	Glycolysis/mitochondrial respiration	Wen et al. (2019)
	Xanthone derivatives	+	—	—	Wang et al. (2018)
	KH2	+	—	Allosteric inhibition of PGAM1	Wang et al. (2024)
	siRNA	+	+	PI3K-AKT-mTOR	Shanske et al. (1987), Park et al. (2021)
PCa	siRNA	+	+	—	Liu et al. (2018c)
OSCC	siRNA	—	+	Paxillin-FAK-SRC CDK4/6 pathway	Liu et al. (2018a)
Leukemia	PGMI-004A	+	—	Glycolysis/PPP flux	Hitosugi et al. (2012)
UBC	shRNA	+	—	—	Peng et al. (2016)

“+” means “positive result”, “-” means “negative result”, “—” means “not research”.

Abbreviations: HCC, hepatocellular carcinoma; NSCLC, Non-small cell lung cancer; PDAC, pancreatic ductal adenocarcinoma; PCa, Prostate cancer; OSCC, oral squamous cell carcinoma; UBC, urothelial bladder cancer.

## 7 The development of PGAM1 inhibitors

PGAM1 is overexpressed in many cancers and mediates tumor growth, migration and invasion. PGAM1 is regarded as a potential target for the treatment of various cancers, and its inhibitors have been developed for cancer therapy (Rigden et al., 1999; Huang et al., 2019a; Li et al., 2017; Wang et al., 2018). Currently reported small-molecule inhibitors of PGAM1 can be divided into two categories: active-site inhibitors and allosteric inhibitors, represented by MJE3, PGMI-004A, KH3, and HKB99, respectively. These inhibitors significant differences in terms of binding sites, chemical structures, interactions with PGAM1, specificity, off-target effects, as well as advantages and disadvantages for clinical translation. The detailed revisions are summarized as follows:

MJE3 is an active-site inhibitor of the small molecule compounds with cellular permeability. It forms a covalent bond with lysine 100 (K100) of PGAM1 through a spiro-epoxide pharmacophore, recognizing the Lys100 (K100) residue in the catalytic pocket of PGAM1, inhibiting its catalytic activity, blocking the conversion of 3-phosphoglycerate to 2-phosphoglycerate, interfering with the glycolytic flux, and exerting its benzyl ester activity after hydrolysis in the cell, effectively inhibiting the proliferation of human breast cancer (MDA-MB-231) and melanoma (MUM2B) (Evans et al., 2007; Evans et al., 2005). Mechanistically, MJE3 irreversibly and completely inhibits PGAM1, showing strong anti-proliferative effects *in vitro*. It has low selectivity and high-dose treatment inhibits PGAM2 in muscles and cardiac tissues, possibly causing myalgia and myocardial glycolytic dysfunction, while it has no binding effect on PGAM4/PGAM5. In addition, MJE3 shows non-specific cysteine covalent modification on various intracellular proteins, with severe on-target/off-target toxicityies, and unsuitable for long-term administration *in vivo*, only used for laboratory research. Only short-term single-dose xenograft experiments were conducted, without long-term toxicity or Patient-Derived Xenograft (PDX) studies (Hitosugi et al., 2012).

Alizarin is the preferred candidate for PGAM1 inhibitors with anti-cancer and antioxidant activities (Balachandran et al., 2021; Xu et al., 2019), showing dose-dependently anti-proliferative activity against human leukemia KG1a cells. Through screening, it was found that Alizarin Red S (ARS) is a more effective PGAM1 inhibitor, with its sulfonic acid group forming electrostatic interactions with K100 and R116 of PGAM1 and the 9,10-anthrone interactions with R90 and R191 through water bridges (Wen et al., 2019). A series of derivatives emerged by adding hydrophobic groups to its sulfonamide bond.

PGMI-004A is an active-site inhibitor derived from alizarin, a dihydroxyanthraquinone sulfonamide derivative. It electrostatically binds to the catalytic active pocket of PGAM1 (residues Arg90, Tyr92, and Arg191), directly competing with the substrate 3-PG for the binding site without forming a covalent bond. PGMI-004A is the first reversible competitive inhibitor of PGAM1 that has been verified to have anti-tumor activity in nude mice, with low toxicity (Wang et al., 2018). However, in tumors with high glycolytic flux, it affects the efficacy due to partial substrate competition; However, its therapeutic efficacy is compromised in tumors with high glycolytic flux due to substrate competition. Furthermore, its poor tissue penetration prevents it from inhibiting the non-enzymatic functions of PGAM1. PGMI-004A exhibits moderate specificity, showing only weak inhibition of PGAM2 at high micromolar concentrations. In the mouse xenograft acute toxicity test, it was confirmed to have extremely low toxicity to normal tissues. The anti-proliferative effect was also verified in H1299 lung subcutaneous nude mice, but no studies were conducted on PDX or immunocompetent animals (Huang et al., 2019b).

HKB99 is an allosteric inhibitor featuring a sulfonamide trans-anthraquinone derivative. It targets a unique binding site located in the C-terminal dimerization loop (residues 201–210) of PGAM1, a region that exhibits poor conservation among PGAM homologous genes. Besides blocking the catalytic activity, HKB99 also locks the monomer conformation of PGAM and disrupts the interaction between PGAM1 and ACTA2. At the same time, it inhibits the enzymatic and non-enzymatic functions. Mechanistically, HKB99 induced the oxidative stress, activated the JNK/c-Jun signaling, and inhibited the protein kinase B (AKT) and extracellular signal-regulated kinase (ERK) pathways. Furthermore, in erlotinib-resistant NSCLC cells, HKB99 was shown to hinder the formation of invasive pseudopodia through the upregulation plasminogen activator inhibitor 2 (PAI-2) (Liang et al., 2021). Among current candidates, HKB99 exhibits the highest selectivity. Its allosteric pocket at the C-terminus is highly specific to PGAM1, resulting in negligible inhibition of PGAM2 and PGAM4, and no cross-interference with PGAM5. In non-small cell lung cancer (NSCLC) PDX models demonstrated favorable pharmacokinetic stability and no observed muscle, liver, or cardiac toxicity following repeated dosing. However, its efficacy has been validated only in preclinical NSCLC, and lacking the broader validation across multi-tumor PDX models (Huang et al., 2019c).

KH3 (Wen et al., 2019) is an N-piperidinequinone derivative that optimizes the structure of Alizarin Red S. Unlike active-site inhibitors, it functions as a non-competitive allosteric inhibitor by binding to a novel allosteric pocket located outside the substrate entry channel of PGAM1, interacting with Arg116, Phe22, and the 109-117 region. It has moderate selectivity, with an extremely low intracellular IC50 (IC50: 0.105 μM), and is effective for pancreatic and hepatocellular carcinoma PDX models, synergizing with anti-PD1 immunotherapy (Yang et al., 2022). It shows moderate cross-reactivity with PGAM2, while having no effect on PGAM4 or PGAM5 function. Systemic toxicity is low in PDX mouse models, with no significant damage to cardiac or skeletal muscle at the therapeutic doses, but long-term high-dose administration may interfere with muscle glycolysis.

**FIGURE 4:**
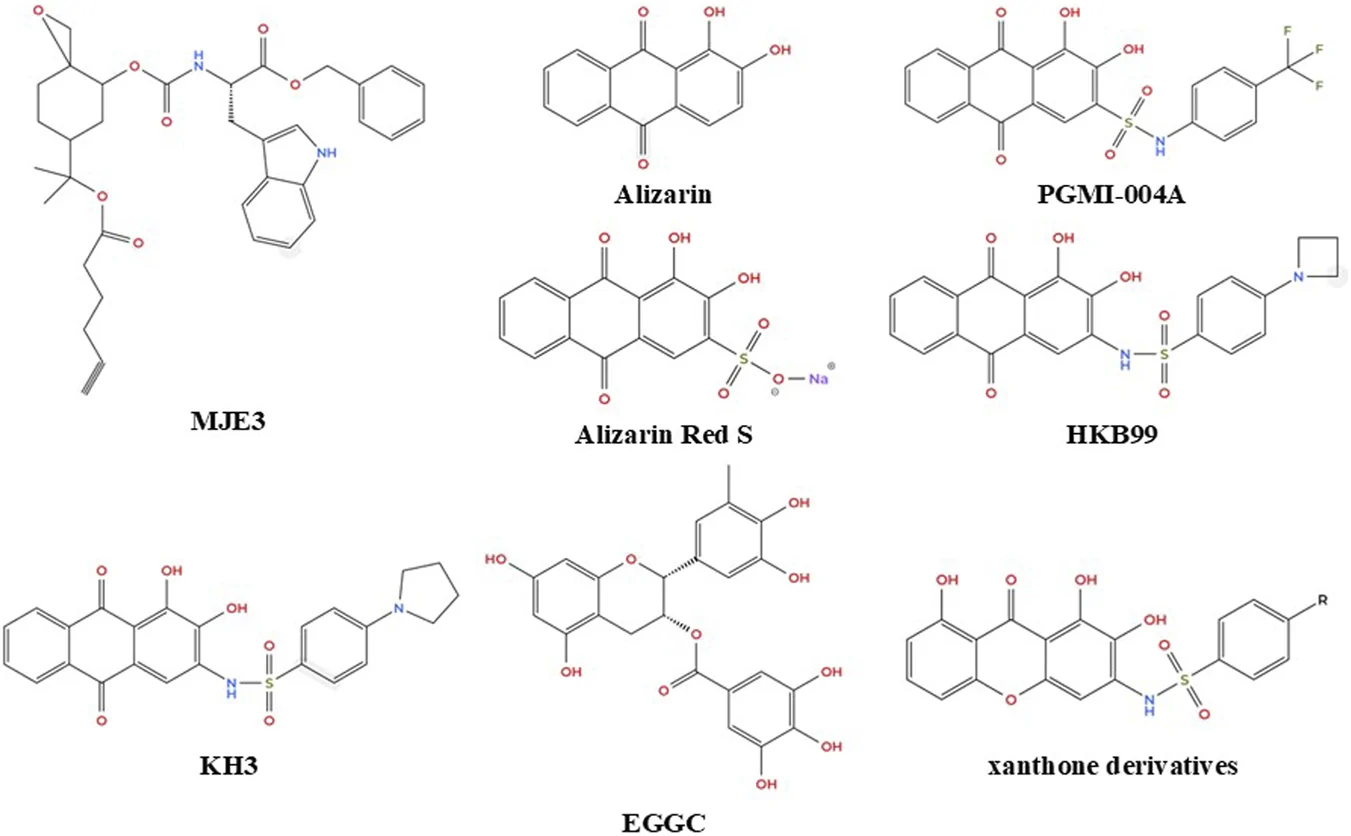
The chemical structures of PGAM1 inhibitors in literatures.

The natural product epigallocatechin-3-gallate (EGCG) extracted from green tea has been identified as a PGAM1 inhibitor. Studies have shown that EGCG inhibits the activity of PGAM1 enzyme by directly damaging glycolysis and PPP in cellulose, without being affected by 3-PG. In addition, EGCG also inhibits the proliferation of cancer cells by regulating the level of 2-PG in cells. However, EGCG could broadly affect multiple signaling pathways, suggesting that EGCG had strong off-targets effects (Figure 4) (Li et al., 2017).

Genetic inhibitors, compared with pharmacological inhibitors, function at the RNA level and generally exhibit more potent anti-cancer. For instance, PGAM1-siRNA or shRNA not only suppress cancer cell proliferation, but also invasion and metastasis (Zhang et al., 2017b; Wen et al., 2018; Zhang et al., 2017a; Liu X. L. et al., 2018). Proteomics analysis revealed that the expression of PGAM1 was significantly elevated in highly metastatic pancreatic cancer cell lines, and silencing PGAM1 could inhibit cell migration and invasion (Liu X. et al., 2018). The study found that the expression of PGAM1 is correlated with the WHO grade of glioma. Knockdown of PGAM1 by siRNA inhibited the proliferation, migration and invasion of U87 glioma cells and promote their apoptosis, with the cell cycle arrested at the S phase (Xu et al., 2016). In PC-3 and 22Rv1 prostate cancer cell lines, Knockdown of PGAM1 by siRNA inhibited cell proliferation, migration and invasion, and enhance cancer cell apoptosis (Wen et al., 2018).

## 8 Conclusion

**FIGURE 5:**
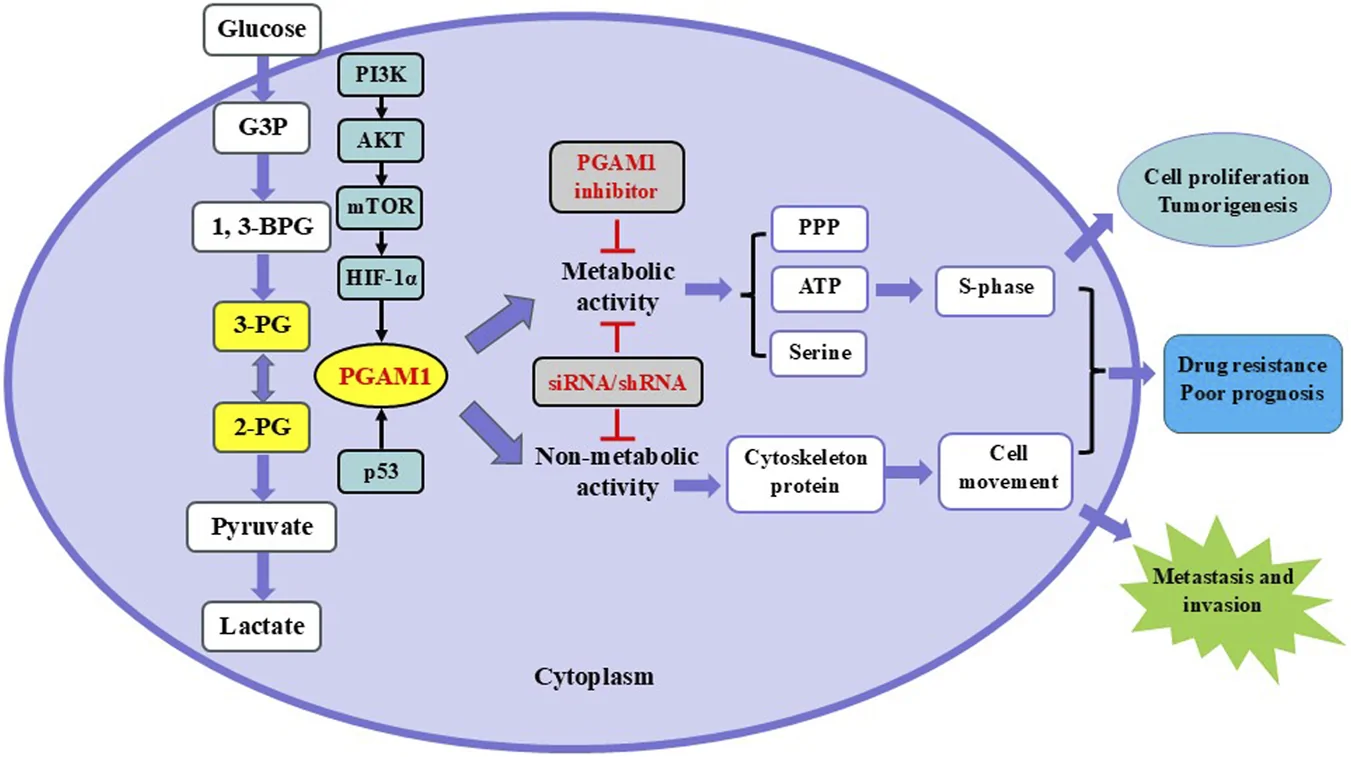
A schematic model shows the current understanding of the role of PGAM1 and the research directions of PGAM1-targeted drugs.

In recent years, significant progress has been made in the study on the biological functions of PGAM1, particularly in aerobic glycolysis and serine synthesis. Increasing evidence indicates that PGAM1 plays a crucial role in the progression of tumors (Figure 5). On the one hand, PGAM1 promotes the proliferation of cancer cells by participating in the glycolytic pathway and regulating the metabolic mode of tumor cells; on the other hand, PGAM1 relies on its unique non-glycolytic functions to enhance the invasion and metastasis ability of cancer cells. Given that PGAM1 is a highly promising and unique anti-cancer target, discovering highly specific PGAM1 inhibitors is of great significance for deeply clarifying its mechanism in tumor progression and evaluating the application value of PGAM1-targeted inhibition strategies in clinical tumor treatment, which is worthy of further research.
